# Inhibition of pulmonary artery smooth muscle cells via the delivery of curcuminoid WZ35 by Cu‐based metal organic frameworks

**DOI:** 10.1049/nbt2.12138

**Published:** 2023-05-16

**Authors:** Zhidan Hua, Mingming Han, Lanlan Song, Yongle Yan, Honglang Chen, Jilong Wang, Chao Li, Yanfan Chen, Hanhan Yan, Mayun Chen

**Affiliations:** ^1^ Department of Pulmonary and Critical Care Medicine The Quzhou Affiliated Hospital of Wenzhou Medical University Quzhou People's Hospital Quzhou Zhejiang China; ^2^ Department of Pulmonary and Critical Care Medicine The First Affiliated Hospital of Wenzhou Medical University Wenzhou Zhejiang China; ^3^ Department of Pulmonary and Critical Care Medicine The Longgang People's Hospital Wenzhou Medical University Wenzhou Zhejiang China; ^4^ Zhejiang Engineering Research Center for Tissue Repair Materials Wenzhou Institute University of Chinese Academy of Sciences Wenzhou Zhejiang China; ^5^ Department of Pulmonary and Critical Care Medicine Ruian People's Hospital The Third Affiliated Hospital of Wenzhou Medical University Wenzhou Zhejiang China

**Keywords:** drug delivery systems, nanobiotechnology, nanoparticles, pulmonary hypertension

## Abstract

Hypoxic pulmonary hypertension (HPH) is a life‐threatening disease that occurs due to a lack of oxygen in the lungs, leading to an increase in pulmonary vascular resistance, right ventricular failure, and ultimately death. HPH is a multifactorial disorder that involves multiple molecular pathways, making it a challenge for clinicians to identify effective therapies. Pulmonary artery smooth muscle cells (PASMCs) play a crucial role in HPH pathogenesis by proliferating, resisting apoptosis, and promoting vascular remodelling. Curcumin, a natural polyphenolic compound, has shown potential as a therapeutic agent for HPH by reducing pulmonary vascular resistance, inhibiting vascular remodelling, and promoting apoptosis of PASMCs. Regulation of PASMCs could significantly inhibits HPH. However, curcumin has the disadvantages of poor solubility and low bioavailability, and its derivative WZ35 has better biosafety. Here, Cu‐based metal organic frameworks (MOF_Cu_) was fabricated to encapsulate the curcumin analogue WZ35 (MOF_Cu_@WZ35) for the inhibition of PASMCs proliferation. The authors found that the MOF_Cu_@WZ35 could promote the death of PASMCs. Furthermore, the authors believed that this drug delivery system will effectively alleviate the HPH.

## Graphical abstract

Cu‐based metal organic frameworks (MOFCu) encapsulate the curcumin analogue WZ35 (MOFCu@WZ35) for the inhibition of PASMCs proliferation.

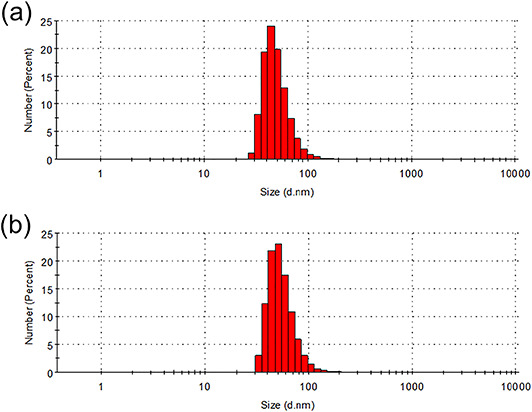

## 1 INTRODUCTION

Pulmonary hypertension (PH) is a rare but serious condition in which there is increased pressure in the blood vessels of the lungs, which can lead to heart failure and death. PH can be caused by a variety of underlying conditions, such as lung disease, heart disease, or genetic factors [1, 2, 3]. One specific subtype of PH is called pulmonary hypertension due to hypoxia, which is characterised by increased resistance in the small arteries of the lungs [4, 5, 6]. HPH is caused by chronic exposure to low oxygen levels, which leads to the narrowing of the pulmonary arteries and an increase in pulmonary vascular resistance. This chronic hypoxia triggers a cascade of molecular and cellular events that result in pulmonary vascular remodelling and right ventricular hypertrophy.

Pulmonary arterial smooth muscle cells (PASMCs) play a key role in the pathogenesis of HPH [7, 8, 9, 10]. In response to chronic hypoxia, these cells undergo changes in their gene expression and become more proliferative and contractile, leading to the thickening of the arterial walls and the reduction of the vascular lumen. The excessive proliferation and migration of PASMCs also contribute to the development of vascular lesions and pulmonary vascular resistance [11, 12, 13]. There is currently no cure for HPH, and the available treatments focus on improving symptoms and slowing down disease progression. Meanwhile, these treatments have limited efficacy and can cause significant side effects. Curcumin and its analogues have recently emerged as promising therapeutic agents for HPH. Curcumin is a natural compound found in the turmeric spice, which has been shown to have anti‐inflammatory, antioxidant, and anti‐proliferative properties [14, 15, 16, 17]. Curcumin and its analogues have been shown to inhibit the proliferation and migration of PASMCs and to reduce pulmonary vascular resistance in animal models of HPH [18]. These compounds also have a favourable safety profile and low toxicity, making them a potential alternative or adjunct to current treatments for HPH. WZ35 is a derivative of curcumin. It has better biological function and its effect on cells is significantly higher than curcumin. The efficacy of WZ35 was better than that of curcumin in the lethal dose via the detection of various cells. However, low bioavailability and potential toxic side effects limit its application in clinical therapy. Nanotechnology is a new research hotspot in recent years, intrinsic good biological properties are widely used in drug delivery research. It can effectively achieve targeted delivery of drugs in the body, reduce the spread of drugs in normal tissues and avoid side effects [19, 20, 21, 22]. Therefore, it will help to improve the delivery efficiency of WZ35 and ensure its suppression effect of PASMCs. By contrast, many other therapeutic vectors and agents are much more complex. Some research have developed nanoparticles of silicium dioxide to intervene the calcium signialling in rat pulmonary artery smooth muscle cells [23]. Meanwhile, some others have developed the liposomal nanoparticles with the encapsulation of iloprost for the treatment in the pulmonary arteries hypertension [24]. However, these studies lack the potential of practical application and cannot avoid the defects of the material carrier itself in application, which is not conducive to the effective role of intracellular drug delivery.

In this study, we prepare the Cu‐based metal organic frameworks to encapsulate the WZ35. The metal organic frameworks have been proved that the good biocompatibility in vivo. In addition, they also exhibit the strong ability of drug loading. We found the cellular uptake of WZ35 was much more via the MOF_Cu_@WZ35. The proliferation of PASMCs were inhibited. The survival rate of PASMCs proved that the delivery system of MOF_Cu_@WZ35 exhibit the strong potential to alleviate the HPH. We believe that this drug delivery system can assist clinical therapy to play a good therapeutic effect.

## 2 MATERIALS AND METHODS

### 2.1 Materials and cells

Dimethylimidazole, methanol, and copper acetate were purchased from JingQiao Bio. Company. The antibodies of DAPI and DCFH‐DA were obtained from the Dakewei Company. The cells of PASMCs were obtained from the rats. The PASMCs were cultured in the hypoxia preconditioning with the Dulbecco’s modified eagle medium (DMEM) containing 10% FBS. All the solution and materials were used according to the instruction.

### 2.2 Preparation and characterisation of MOF_Cu_@WZ35 nanoparticles

The metal organic frameworks were fabricated through the methods published before. 5 mg dimethylimidazole was dissolved in 10 mL methanol. One milligram of copper acetate was dissolved in 5 mL methanol. Then, 2.5 mL of copper acetate solution was dropped into the 5 mL dimethylimidazole solution and further blending at 37°C for 30 min. Finally, the prepared MOF_Cu_ were centrifugated at 8000 rpm for 20 min. The collected nanoparticles were washed three times with the methanol. The purified nanoparticles were stored at 4°C for usage. The diameter of nanoparticles were determined by the dynamic light scattering.

For the preparation of MOF_Cu_@WZ35 nanoparticles, the WZ35 was inserted into the mixed solution during the preparation. The purification methods were the same as the MOF_Cu_. The diameter of MOF_Cu_@WZ35 nanoparticles were also measured by the dynamic light scattering. Meanwhile, we determined the morphology of MOF_Cu_@WZ35 via the transmission electron microscopy.

### 2.3 Cellular uptake in vitro

In order to verify the target ability of MOF_Cu_ to the PASMCs, we labelled the MOF_Cu_ with Rhobmine B. Therefore, the nanoparticles could be indicated with red fluorescence, which could be visualised in the cell. By comparison, we use Rhobmine B as the control group. The fluorescence labelled nanoparticles and free dye were incubated with PASMCs (cultured in the hypoxia preconditioning) for 8 h. Then, the cells were washed with PBS for three times. Subsequently, the cytoskeleton was stained with green phalloidine. Meanwhile, the cell nucleus were indicated with 0.1% DAPI. Then, the slices were mounted on the glass with anti‐fluorescence quencher. Finally, they were observed with the laser scanning confocal microscope.

### 2.4 In vitro cell viability

The proliferation of PASMCs could be affected by the treatment of MOF_Cu_@WZ35. Therefore, we incubated the cells with different concentration of MOF_Cu_@WZ35 to determine the effect of inhibition on the cell proliferation. The cell viability of PASMCs was measured by the CCK8 kit. The cell survival rate was calculated with the comparison of control group.

## 3 RESULTS AND DISCUSSION

**FIGURE 1:**
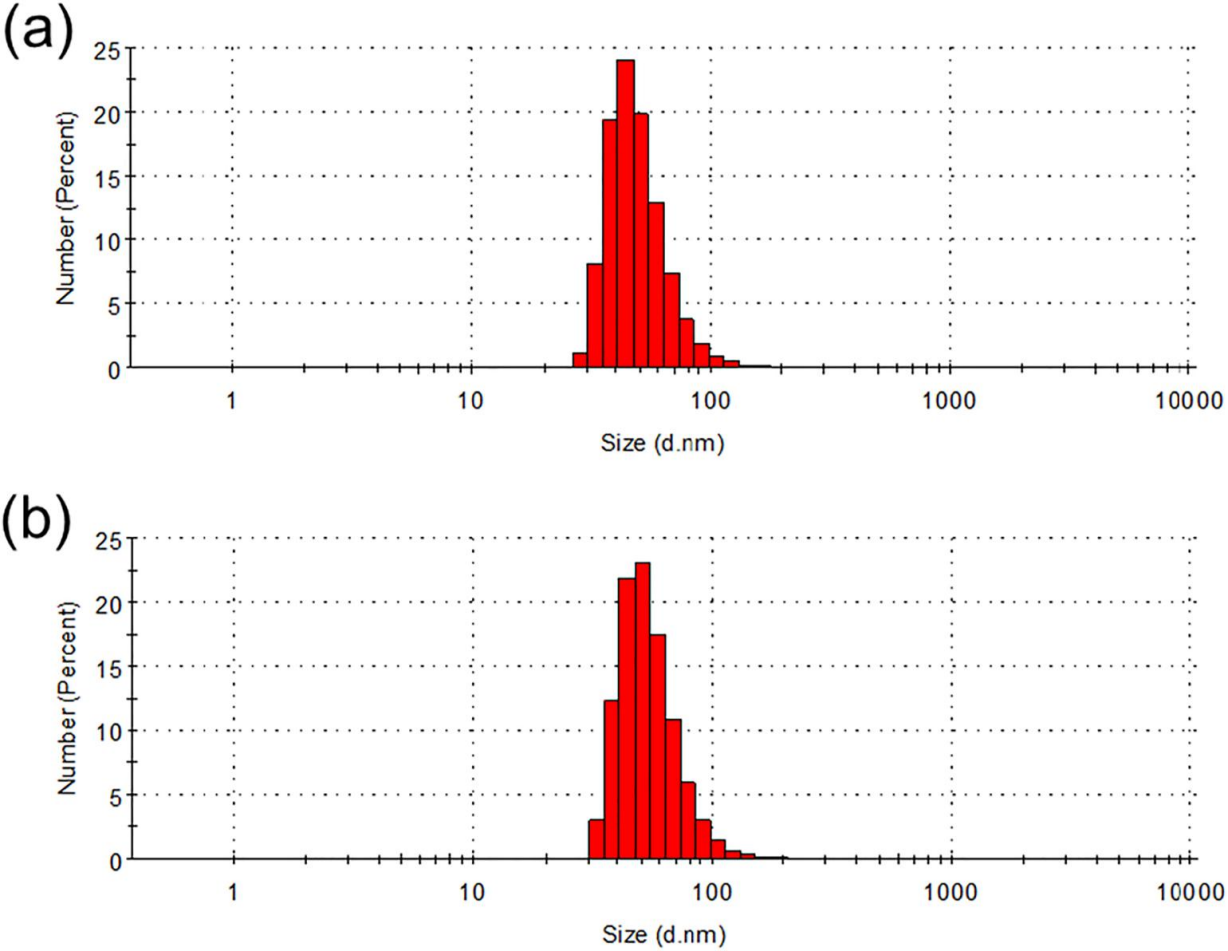
The diameter of nanoparticles. The diameters of (a) MOF_Cu_ and (b) MOF_Cu_@WZ35 measured by the Zetasizer Nano ZS.

The WZ35 nanoparticles were synthesised using a nanoprecipitation method. Briefly, WZ35 and dimethylimidazole were dissolved in methanol, and the solution of copper acetate was added dropwise to the mixed solution under stirring. The resulting mixture was centrifuged to remove any aggregates and methanol. The size of the nanoparticles were measured using a Zetasizer Nano ZS, and the morphology was examined using transmission electron microscopy (TEM). The results in Figure 1 showed that the MOF_Cu_ exhibited a diameter of ∼116 nm (Figure 1a), while the diameter of MOF_Cu_@WZ35 was around 119 nm (Figure 1b).

**FIGURE 2:**
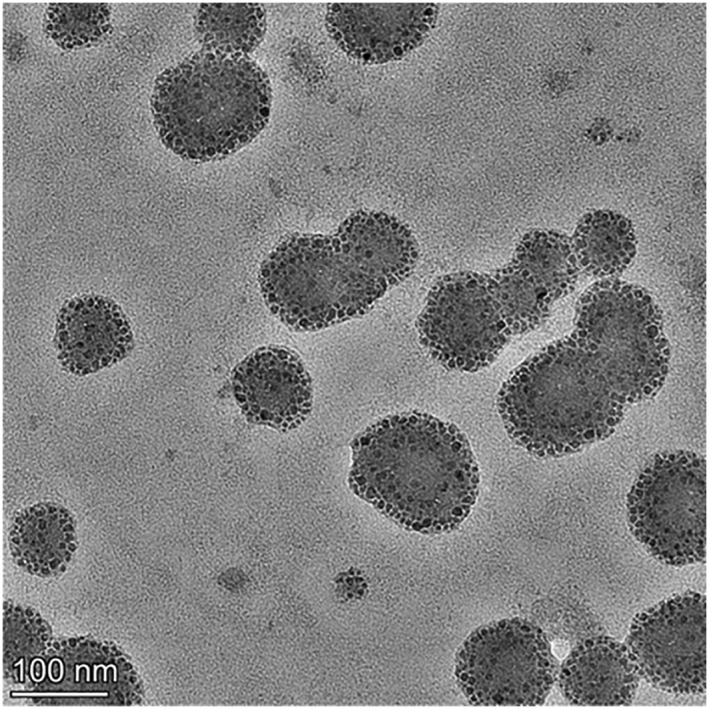
The image of MOF_Cu_@WZ35 detected by the transmission electron microscopy.

After the characterisation, the fabricated MOF_Cu_@WZ35 was further analysed for the analysis of morphology. The prepared nanoparticles were formulated at the concentration of 1 mg/mL and dispersed by ultrasonic to ensure uniformity. Then, the prepared MOF_Cu_@WZ35 nanoparticles were dropped on the copper screen. Excess liquid was removed with absorbent paper and placed in an oven to dry the sample. Then, they were detected by the transmission electron microscopy. It could obviously be seen that the nanoparticles exhibit a spherical structure (Figure 2). The measurements indicated that the diameter of the nanoparticles was all around 100 nm. In addition, the picture also showed some aggregates inside the nanoparticles. These black dots were present in all nanoparticles. Therefore, we speculated it may be the drugs of WZ35 that were contained inside of the MOF_Cu_.

**FIGURE 3:**
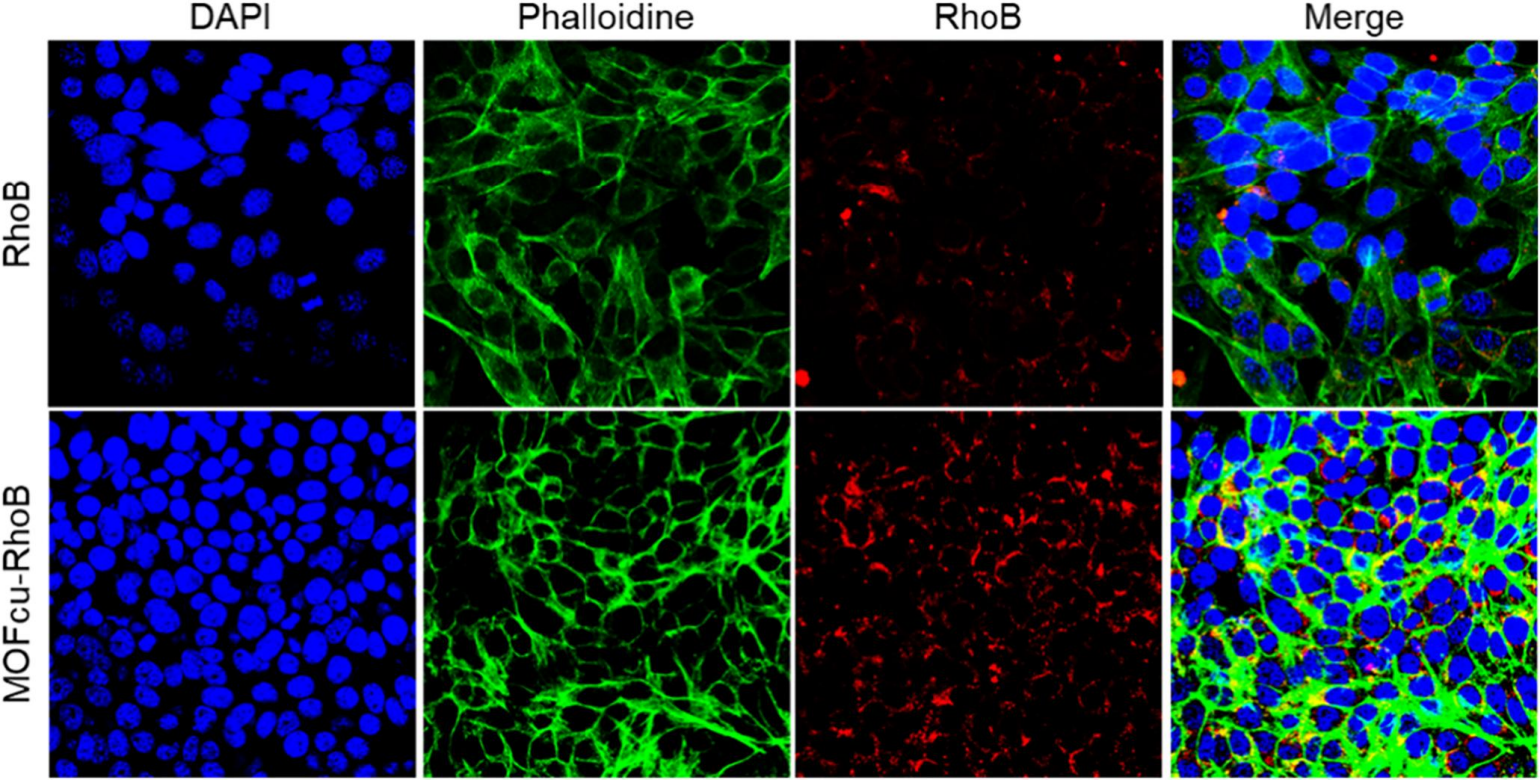
The cellular uptake of MOF_Cu_‐RhoB and RhoB detected by the laser scanning confocal microscope.

As we all know, nanoparticles have significant advantages in drug delivery. Here, we aim to prove that the MOF_Cu_ could deliver more drugs inside the cells. To visualise the effects of drug delivery via the MOF_Cu_, we decided to use the fluoresce dye of Rhomine B to label the nanoparticles. Then, the red nanoparticles of MOF_Cu_‐RhoB were incubated with the PASMCs. After the incubation, the cells were collected and stained with DAPI and phalloidine. They were detected by the laser scanning confocal microscope. We found that the red fluoresces in MOF_Cu_‐RhoB group was much stronger than that of RhoB group (Figure 3). The results demonstrated that the MOF_Cu_ could deliver more payloads to the cells.

**FIGURE 4:**
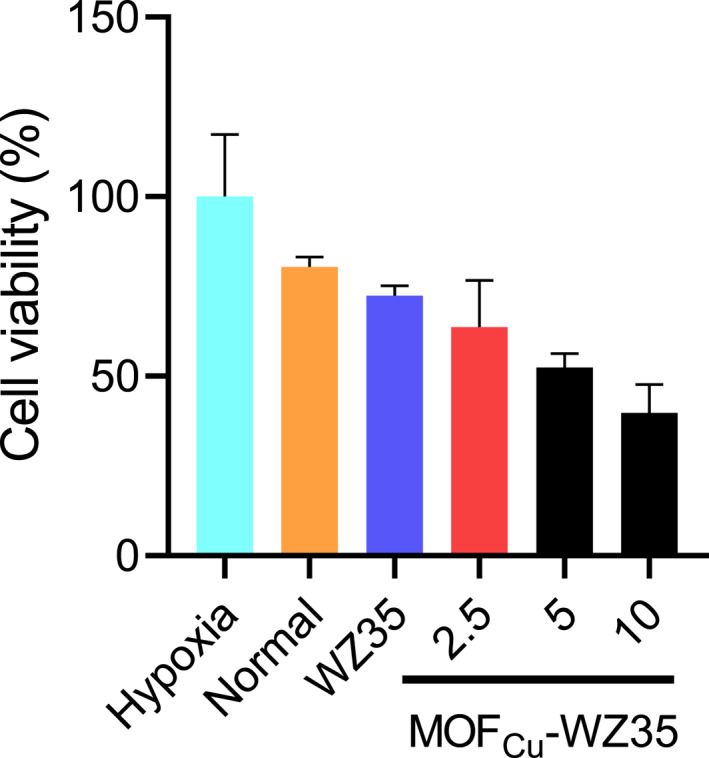
The cell viability in PASMCs triggered by the MOF_Cu_@WZ35.

After exploring the underlying mechanism, we further examined whether there was an effect on the cell survival. The cells of PASMCs were incubated with free WZ35 and different concentration of MOF_Cu_@WZ35 at the hypoxic microenvironment. As the HPH was induced by malignant proliferation of cells under hypoxic conditions. Meanwhile, we also selected the PASMCs cultured at the normal oxygen conditions for the control. The other cells were cultured in the hypoxic microenvironment, which keeps the potential of HPH disease. From the result, we found that the cell viability of MOF_Cu_@WZ35 was lower than that of the control group and free WZ35 group. In addition, we found that the cell viability in MOF_Cu_@WZ35 group was lower and that accompanies the increase of WZ35 content (Figure 4). Therefore, the result demonstrated that the nanoparticle could assist the drug delivery into the target cells. These results confirm that this nano‐drug delivery system can effectively inhibit abnormal PASMCs proliferation under hypoxic conditions and has the potential to be used in the treatment of HPH.

## 4 CONCLUSION

In summary, HPH is a serious and progressive disease that is caused by chronic exposure to low oxygen levels. PASMCs play a key role in the pathogenesis of HPH, and current treatments have limited efficacy and significant side effects. Curcumin and its analogues represent a promising therapeutic approach for HPH. This study demonstrates that WZ35 nanoparticles inhibit the proliferation of rat PASMCs, promote apoptosis, and have the potential to reduce HPH in rats. The development of WZ35 nanoparticles as a therapeutic agent for HPH could potentially provide a promising treatment option for patients with HPH. This study provides a foundation for further research on the use of WZ35 nanoparticles in the treatment of HPH and other diseases. Absolutely, further research is needed to fully evaluate their potential in the clinic.

## AUTHOR CONTRIBUTIONS

Zhidan Hua and Mayun Chen conceived the idea and designed the experiments, Mingming Han wrote the original draft. Lanlan Song and Jilong Wang performed the experiments and analysed the data. Yongle Yan and Honglang Chen helped to analyse the data and provided valuable advice. Chao Li, Yanfan Chen and Hanhan Yan co‐wrote the manuscript. All the authors read and approved the final manuscript.

## CONFLICT OF INTEREST STATEMENT

The authors confirm that there are no conflict of interest.

## Data Availability

The data that support the findings of this study are available on request from the corresponding author. The data are not publicly available due to privacy or ethical restrictions.
